# ECOALIM: A Dataset of Environmental Impacts of Feed Ingredients Used in French Animal Production

**DOI:** 10.1371/journal.pone.0167343

**Published:** 2016-12-08

**Authors:** Aurélie Wilfart, Sandrine Espagnol, Sylvie Dauguet, Aurélie Tailleur, Armelle Gac, Florence Garcia-Launay

**Affiliations:** 1 UMR SAS, INRA, Agrocampus Ouest, Rennes, France; 2 IFIP, Institut du porc, Le Rheu, France; 3 Terres Inovia, Pessac, France; 4 ARVALIS-Institut du végétal, La Chapelle Saint Sauveur, France; 5 Institut de l’Elevage, Monvoisin, Le Rheu, France; 6 UMR PEGASE, INRA, Agrocampus Ouest, Saint-Gilles, France; Universitat Politècnica de València, SPAIN

## Abstract

Feeds contribute highly to environmental impacts of livestock products. Therefore, formulating low-impact feeds requires data on environmental impacts of feed ingredients with consistent perimeters and methodology for life cycle assessment (LCA). We created the ECOALIM dataset of life cycle inventories (LCIs) and associated impacts of feed ingredients used in animal production in France. It provides several perimeters for LCIs (field gate, storage agency gate, plant gate and harbour gate) with homogeneously collected data from French R&D institutes covering the 2005–2012 period. The dataset of environmental impacts is available as a Microsoft^®^ Excel spreadsheet on the ECOALIM website and provides climate change, acidification, eutrophication, non-renewable and total cumulative energy demand, phosphorus demand, and land occupation. LCIs in the ECOALIM dataset are available in the AGRIBALYSE^®^ database in SimaPro^®^ software. The typology performed on the dataset classified the 149 average feed ingredients into categories of low impact (co-products of plant origin and minerals), high impact (feed-use amino acids, fats and vitamins) and intermediate impact (cereals, oilseeds, oil meals and protein crops). Therefore, the ECOALIM dataset can be used by feed manufacturers and LCA practitioners to investigate formulation of low-impact feeds. It also provides data for environmental evaluation of feeds and animal production systems. Included in AGRIBALYSE^®^ database and SimaPro^®^, the ECOALIM dataset will benefit from their procedures for maintenance and regular updating. Future use can also include environmental labelling of commercial products from livestock production.

## Introduction

The Food and Agriculture Organization (FAO) stated that livestock farming systems are involved in 14.5% of human-induced greenhouse gas (GHG) emissions [1]. Recent studies investigated ways to provide protein to feed the increasing world population by 2025 while also decreasing environmental impacts of agricultural production. Billen *et al*. [2] stated that a change in human diets in several regions of the world and moderate agricultural intensification in all regions of the world may reduce international trade, improve food sovereignty and reduce nitrogen (N) pollution. In contrast, the FAO argued that no technically or economically viable alternative exists for intensive animal production to feed the world [3]. Although there is a clear debate on how to provide an adequate supply of nutrients to the world population by 2050, the scientific community agrees on the need to decrease pollutant emissions related to livestock production; consequently, it focuses on estimating environmental impacts of livestock production reliably enough to identify mitigation options [4].

Life cycle assessment (LCA) is the most popular and recognised methodology, with an ISO standard [5], to estimate environmental impacts of animal products [6, 7]. In pig and poultry systems, feed production contributes greatly to environmental impacts of animal products. In particular, it accounts for 50–85% of climate change impact, 64–97% of eutrophication potential, 70–96% of energy use and nearly 100% of land occupation [8–13], with differences among animal products (e.g. broiler vs. layer), type of production systems (e.g. industrial, backyard) and geographic location [14]. In dairy production, methane from enteric fermentation is the main GHG-emission source, but feed production accounts for approximately 36% of GHG emissions, with differences among geographic regions of the world [15]. In beef production, feed production can contribute up to 55% of GHG emissions [16]. Therefore, given the large contribution of feed production to the environmental load of livestock products, it is necessary to implement mitigation strategies based on robust and accurate data on environmental impacts of feed ingredients.

Environmental impacts of feeds are influenced mainly by their ingredients. Some of these ingredients account for more than 10% of feed composition and have relatively high impacts on resulting rations. For instance, soya bean meal imported to France is usually incorporated at levels of 10% and 22% in pig [17] and poultry growing feeds [18], respectively, and has relatively high potential climate change impacts, related to deforestation in the past 20 years in the soya bean production area of west-central Brazil [19]. Other feed ingredients are incorporated in small amounts into feeds but have high environmental impacts per kilogram, e.g. amino acids and monocalcium phosphate [17]. From a nutritional viewpoint, feed ingredients are classified into cereals and cereal co-products (mainly to supply energy), protein-rich crops and oilseeds (mainly to supply protein), by-products from industry (mainly to supply fibre), and additives (mainly to achieve balance among amino acids and improve phosphorus (P) digestibility). Normally, feed formulation aims to reduce input costs under nutritional constraints, without considering environmental impacts of the feed ingredients incorporated. Feed formulation based on environmental criteria can promote substitutions among feed ingredients within these categories, with a potential difference in feed cost and environmental impacts.

To formulate low-environmental impact feeds, it is necessary to incorporate data on environmental loads of feed ingredients into the feed formulation program. One alternative is to use a dataset based on consistent perimeters and methodologies for LCA. Bertoluci *et al*. [20] emphasised that combining data from heterogeneous studies with different assumptions and perimeters can compromise the consistency of the dataset and the relevance of conclusions. Currently, few international databases include environmental impacts of feed ingredients [21, 22]. The ecoinvent^®^ database relies mainly on Swiss data and practices for the life cycle inventories (LCIs) of agricultural production and provides only a few feed-ingredient LCIs. The European database Agri-Footprint [21] contains standardised data (LCIs and environmental impacts) for several countries but not for all feed ingredients (e.g. feed ingredients processed in France, amino acids). Using it to formulate feeds in France would require incorporating data from other sources for missing feed ingredients, thus increasing the uncertainty in results. Agri-Footprint is also mainly intended as a source of secondary or background data for LCI or LCA of animal products from the perspective of environmental labelling of food products [21]. It is thus more appropriate for comparing animal production systems and mitigation options at the level of the animal unit subsystem. Consequently, an LCA database dedicated to feed formulation is needed, which requires greater data accuracy and model complexity for LCAs of feed ingredients [23].

Feed formulation in France involves mostly French feed ingredients, which result from several cropping systems under a variety of soil and climate conditions and cropping practices. A previous study [18] highlighted the need for homogeneously developed data on environmental impacts of feed ingredients to formulate low-impact feeds and investigate mitigation options.

The ECOALIM dataset is the result of a collaborative project between research and extension institutes, with the participation of animal-nutrition companies. ECOALIM collects the most accurate and representative data available to date for LCIs of French feed ingredients. This dataset can be used in France and in countries importing French feed ingredients. This article presents the methodological choices and perimeters implemented to develop the ECOALIM dataset, as well as its potential application.

## Materials and Methods

LCA is a methodology which calculates environmental impacts of a specific product by including all the resources needed to produce it and all pollutant emissions associated with its life cycle (from extraction of raw materials to recycling of the product’s disposal or recycling). An LCA follows 4 steps defined by an ISO standard [5]: goal and scope definition, inventory analysis, impact assessment, and interpretation of results.

### Goal and scope definition

**Fig 1:**
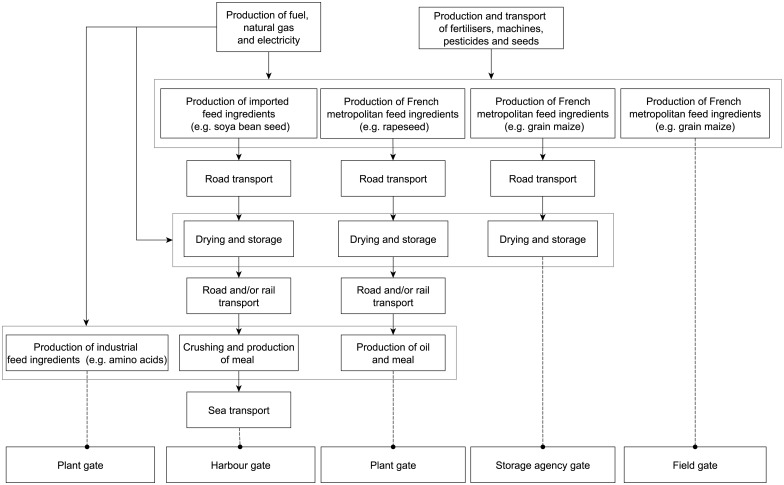
Flow diagram for production of the feed ingredients included in the ECOALIM dataset, with main processes for crop-input production, crop production and feed-ingredient production. System perimeters include all sub-processes.

For the ECOALIM dataset, several system perimeters were defined (Fig 1): field gate, storage-agency gate, plant gate, and harbour gate. The field gate is relevant for assessing impacts of on-farm feed production (i.e. crops produced and directly used on-farm). Feed formulation by feed companies requires additional perimeters: plant gate for the co-products of cereals, oilseeds and protein crops (e.g. meals) as well as for industrial products (e.g. amino acids) processed in France; storage-agency gate for cereals (perimeter includes grain drying); and harbour gate for imported feed ingredients.

The impacts considered were climate change with (CCLUC) or without land-use change (CC), eutrophication (EU), acidification (AC), land occupation (LO), non-renewable (CEDNR) and total energy demand (CEDTOT) and P demand (PD). PD was included to account for the non-renewable P resource incorporated in fertilisers and feeds. The other impact categories are usually included in agricultural LCAs.

When a production process generates multiple final co-products, it is necessary to allocate the process’s impacts to the co-products. In ECOALIM, impacts of co-products of cereals and maize, as well as oils and meals, were calculated using economic allocation. To perform this allocation based on the relative economic value of each co-product, the Olympic five-year (2008–2012) average price of each co-product was calculated. The functional unit used is kilogram of feed ingredient at the reference water content, which is the usual functional unit for feed ingredients.

### Life cycle inventories

#### French crops

Average LCIs for the following French crops were included in the ECOALIM dataset: maize, wheat, barley, oat, triticale, sorghum, rapeseed, sunflower, faba bean, flaxseed, pea, and soya bean (Table 1).

**Table 1:** Main inputs used and dry matter yield for French crops used as ingredients in animal feed.

Name \ Unit	N mineral	N^a^ Manure	P_2_O_5_ (mineral + manure)	K_2_O (mineral + manure)	Seed	Pesticide (active ingredient)	Diesel	Agricultural machinery	Irrigation water	Moisture content at harvest	Yield (dry matter)
	kg/ha	kg/ha	kg/ha	kg/ha	kg/ha	kg/ha	kg/ha	kg/ha	m^3^/ha	%	kg/ha
Wheat FR	163	11	25+7	42+12	137	1.87	77	6.7	15	15	7100
Grain maize FR	176	37	45+25	40+54	28	2.03	83	8	645	28	10,672
Barley FR	135	15	33+4	33+14	128	2.5	72	7	9	15	6722
Silage sorghum FR	15	45	18+13	18+65	9	2.3	135	12.2	120	100	10,700
Grain sorghum FR	60	0	48+10	27+0	9	2.3	80	7.2	120	15	5800
Oat FR	110	30	30+7	28+0	97	1.7	63	6	0	15	4900
Triticale FR	120	11	23+41	11+23	120	1.24	62	6	0	15	5200
Pea FR	0	9	22+7	19+10	219	4.2	92.5	8.6	50	15	3910
Faba bean FR	0	8	46+20	31+30	20.6	3.05	81	7.9	0	15	4309.5
Rapeseed FR	161,6	17	38+10	28+0	2.5	1.98	78.3	8.2	0	9	2951
Sunflower FR	40.5	11	25+7	25+0	4.3	1.67	77.4	7.7	24	9	2169
Soya bean FR	1.5	9	24+9	28+0.3	91.2	1.39	62	6	750	14	2683
Flaxseed FR	84	6	33+2	24+20.7	35	0.25	81.5	7.3	0	10	1823

^a^ amount of N from manure after allocation among crops in rotation

LCIs for French crops were based on those in the French national database, Agribalyse^®^, for the main agricultural products [24], with updated emission factors for ammonia [25] and references for agricultural practices, when available. One major improvement compared to Agribalyse^®^ is the distribution of the impacts associated with P fertilisation and nitrate leaching among the crops in the same crop rotation (according to crop requirements, P uptake and nitrate leaching among crops).

For all French crops, average LCIs (yields; amounts of fertilisers, pesticides and seeds) were obtained from French agricultural data [26–28] (Table 2). For maize, wheat, barley, rapeseed and sunflower, specific LCIs were constructed for systematic cover cropping, systematic organic fertilisation, and introduction of a protein crop in the crop rotation, based on data from the farm network of the French agricultural institute dedicated to cereals (ARVALIS-Institut du végétal), to assess variability in the results due to crop management.

**Table 2:** Data references for average life cycle inventories for the main French crops.

Crop	Inventory source	Yield / average national inventory	Organic fertilisation	Other inputs (mineral fertiliser, seeds, agricultural machinery, etc.)
Rapeseed	AGRIBALYSE^®^	Agreste^a^ 2006–2010	Agreste survey 2006^b^	Terres Inovia surveys 2008–2010 and expert knowledge
Sunflower	AGRIBALYSE^®^	Agreste^a^ 2006–2010	Agreste survey 2006^b^	Terres Inovia surveys 2006 and 2009
Pea	AGRIBALYSE^®^	Statistical data^c^ from UNIP^d^-ARVALIS^e^2005–2009	Agreste survey 2006^b^ + expertise	Agreste survey 2006^b^ and expert knowledge
Faba bean	AGRIBALYSE^®^	Statistical data^c^ from UNIP^d^-ARVALIS^e^ 2005–2009	Expert knowledge	Expert knowledge
Flaxseed	AGRIBALYSE^®^	Agreste^a^ 2005–2009	Agreste survey 2006^b^ and expert knowledge	2013 National survey by Terres Inovia and expert knowledge
Soya bean	ECOALIM	Agreste^a^ 2008–2012	Agreste survey 2006^b^ and expert knowledge	2012 National survey by Terres Inovia and expert knowledge
Soft wheat	AGRIBALYSE^®^	Agreste^a^ 2005–2009	Agreste survey 2006^b^	Agreste survey 2006^b^
Maize	AGRIBALYSE^®^	Agreste^a^ 2005–2009	Agreste survey 2006^b^	Agreste survey 2006^b^
Barley	AGRIBALYSE^®^	Agreste^a^ 2005–2009	Agreste survey 2006^b^	Agreste survey 2006^b^
Sorghum	ECOALIM	Agreste^a^ 2008–2012	Expert knowledge	Expert knowledge, Arvalis experimental farm network 2008–2012
Oat	ECOALIM	Agreste^a^ 2008–2012	Expert knowledge	Expert knowledge, Arvalis experimental farm network 2008–2012
Triticale	AGRIBALYSE^®^	Agreste^a^ 2005–2009	Expert knowledge	Expert knowledge

^a^Online database (in French) available at: http://www.agreste.agriculture.gouv.fr/enquetes/statistique-agricole-annuelle-saa/

^b^Agreste, 2006. Enquête sur les pratiques culturales en 2006. Available from: http://agreste.agriculture.gouv.fr/publications/chiffres-et-donnees/article/enquete-sur-les-pratiques

^c^Online database (in French) available at: http://www.unip.fr/marches-et-reglementations/statistiques-france/surfaces-rendements-et-productions.html

^d^UNIP: French agricultural technical institute dedicated to legume crops

^e^ARVALIS: French agricultural technical institute dedicated to crops

#### Non-French crops

Average LCIs of the following non-French crops were included in the dataset: Brazilian soya bean; US soya bean, sorghum, and maize; UK wheat; Malaysian palm oil tree; Pakistani sugarcane; and Ukrainian sunflower (Table 3). Data for resources came from scientific publications [19, 22, 29] and statistics from national databases [30–33] or FAOSTAT [34]. Resources and emissions in LCIs were calculated according to previously published methodologies [19, 35].

**Table 3:** Main inputs used and dry matter yield for European and non-European crops serving as feed ingredients for animal feed (BR: Brazil, US: United States of America, UK: United Kingdom, MY: Malaysia, PK: Pakistan, UA: Ukraine).

Crop \ Unit	N mineral	N manure	P_2_O_5_ (Mineral +manure)	K_2_O (Mineral +manure)	CaO	Seed	Pesticide (active ingredient)	Diesel	Agricultural machinery	Irrigation water	Moisture content at harvest	Yield (dry matter)
	kg/ha	kg/ha	kg/ha	kg/ha	kg/ha	kg/ha	kg/ha	kg/ha	kg/ha	m^3^/ha	%	kg/ha
Soya bean BR	5.5	1.3	80 +0.5	80+0.5	518	53	1.7	76.0	18.0	0	18	2708
Soya bean US	18.5	0	52+0	88+0	0	72.5	5.4	53.4	4.9	5	14	2411
Wheat UK	188	0	17+0	23+0	0	200	8.6	62.6	5.6	0	15	6715
Sorghum US	81.6	0	31+0	24+0	0	1.3	4.0	79.8	7.3	838	14	3354
Maize US	156	0	66+0	93+0	0	20.3	4.1	80.4	7.5	871	28	6732
Palm oil tree MY	157	0	32	236	43	-	1.7	26	67,119.0	448	47	24,978
Sugarcane PK	125	29	130+19	0+24	0	-	8.25	98.2	-	11616	70	15,090
Sunflower UA	28.8	0	15+0	15+0	0	3.84	2.04	75.1	7.1	0	22	1432

#### Industrial products

Industrial products in the dataset include products derived from crops and products that result from chemical synthesis, fermentation, mining extraction, and animal by-product processing. Co-products directly derived from crops are oils, meals, Dried Distilled Grains with Solubles (DDGS), molasses, pulps, and flours associated with extrusion processes, crushing, milling, distillation, etc. Products secondarily derived from crops are starch and gluten feeds. Processing factories were surveyed to collect the relevant underlying data in 2013–2014, except for French oils and meals and non-French feed ingredients [36]. For other industrial products, LCIs are from Garcia-Launay *et al*. [17] for amino acids, confidential industrial data for minerals, the French Technical Centre for Meat (ADIV) data for animal by-products, and ecoinvent data adapted to the French context for vitamins [36].

### Calculation of emissions

Calculations of gaseous emissions (NH_3_, NO_x_, N_2_O, and CO2) and emissions to soil and water (NO_3_^-^, PO_4_^3-^, heavy metals) were based on the AGRIBALYSE^®^ method [24]. Briefly, NH_3_ emissions came from EMEP/EEA 2013 Tier 2 [25] and N_2_O emissions from IPCC Tier 1 [37]. Phosphate emissions were calculated based on previously developed models for soil loss [38] parametrised for France and the SALCA-P model [22] for P emissions into water (i.e. leaching, run-off, erosion). NO_x_ emissions were calculated according to EMEP/EEA 2013 Tier 1 [25]. Nitrate emissions were calculated according to a spatial grid of leaching risk based on the crop and the regional area from COMIFER [39] adapted by Tailleur *et al*.[40]. Gaseous emissions from agricultural machinery combustion were estimated with the method of Nemecek and Kägi [22]. CO_2_ emissions from urea fertilisation and lime application were estimated from IPCC Tier 1 [38]. Heavy metal emissions were estimated from the SALCA-SM model adapted to the French context [41, 42].

### Impact assessment

ECOALIM uses the ILCD characterisation method recommended by the Joint Research Centre [43], as well as the CML-IA characterisation method [44], which is the most popular in agricultural LCA. Energy demand is calculated according to the CED 1.08 method [44].

CC and CCLUC are expressed in kg CO_2_-eq, AC in both molc H^+^-eq (AC_ILCD_) and kg SO_2_-eq (AC_CML_), terrestrial EU in molc N-eq, freshwater EU in kg P-eq, marine EU in kg N-eq, EU_CML_ in kg PO_4_^3-^ eq, LO in m².y, and CEDNR and CEDTOT in MJ. PD is expressed in kg P and includes all P and phosphate inputs throughout the life cycle. Calculations were performed using SimaPro^®^ software v8.0.5.13 (PRé Consultants, Amersfoort, The Netherlands) and the attributional ecoinvent v3.1 database for background data [36].

### Statistical analysis

The dataset was subject to a series of multivariate analyses using R statistical software [45] to describe variability in the impacts associated with each type of feedstuff. From all of the impact categories available in the dataset, we selected CCLUC, AC_CML_, EU_CML_, CEDNR, PD, and LO after examining the correlation coefficient between each pair of impacts and excluding impacts that were highly correlated. To address the high skewness of impact distributions, the quantitative variables CCLUC, AC_CML_, EU_CML_, CEDNR, PD, and LO were converted into qualitative variables with levels corresponding to the 4 quartiles of the distribution of each impact. The feed-ingredient typology was constructed with the active qualitative variables CCLUC, AC_CML_, EU_CML_, CEDNR, PD, and LO and the illustrative qualitative variable “Feedstuff Type” using multiple correspondence analysis (MCA), followed by hierarchical clustering (HC) on the 5 first dimensions of the MCA. This method classified feed ingredients that are similar to each other but different from others by maximising intra-group homogeneity and inter-group diversity. After plotting the inertia of the classifications as a function of the number of classes, we chose a number of classes that did not result in a gap in inertia. Finally, levels of active variables, the illustrative variable, as well as the classes of the typology, were plotted on the factor map.

## Results and Discussion

The objectives of this collaborative work among institutes were to develop a dataset of LCIs and environmental impacts of feed ingredients used for French livestock nutrition. This section addresses the content of the dataset, illustrates the typology of the categories of environmental impacts of feedstuffs available in it, and discuss its potential uses.

### Dataset description

**Fig 2:**
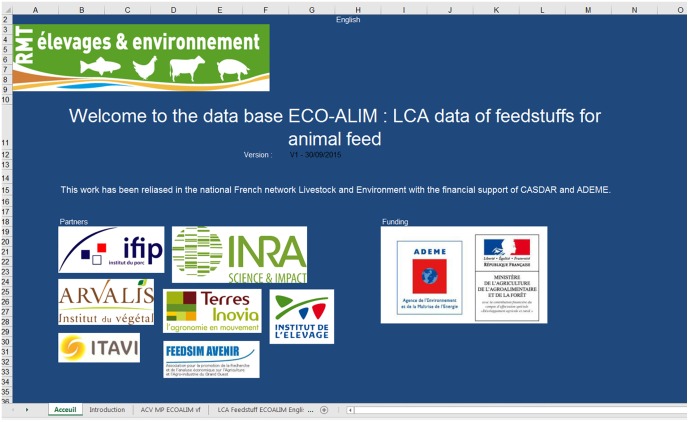
Overview of the ECOALIM impact dataset in Microsoft^®^ Excel, freely available at http://www6.inra.fr/ecoalim_eng/ and from the Dryad Digital Repository: http://dx.doi.org/10.5061/dryad.14km1.

The ECOALIM dataset contains LCIs for 149 average feed ingredients and 16 feed ingredients from specific crop-management practices (Table 4). It offers a wide range of feedstuffs used in several kinds of livestock production (cattle, pig and poultry) and different perimeters for LCA, for feed formulation by feed manufacturers, farm cooperatives and farmers (on-farm). A Microsoft^®^ Excel file is available online (Fig 2) which shows the partners involved and provides an overview of the environmental impacts available by default (CCLUC, AC_ILCD_, EU_CML_, CEDNR, PD, LO_CML_) in the dataset.

**Table 4:** Number of life cycle inventories available in the ECOALIM dataset according to the type of feed ingredient and perimeter.

Type of feed ingredient	Perimeter			
	At field	At French harbour	At mill or processing plant	At storage agency
Cereals	8	2	0	17
Co-products from food industry	0	0	2	0
Co-products of maize	0	1	5	0
Co-products of wheat	0	0	5	0
Fats	0	6	22	0
Industrial amino acids	0	0	5	0
Minerals	0	0	10	0
Oil seeds and protein crops	6	0	4	13
Oil meals	0	7	22	0
Other co-products of animal origin	0	0	2	0
Other co-products of plant origin	1	1	4	0
Silage	5	0	0	0
Vitamins	0	0	1	0

### Typology of the feed ingredients

**Fig 3:**
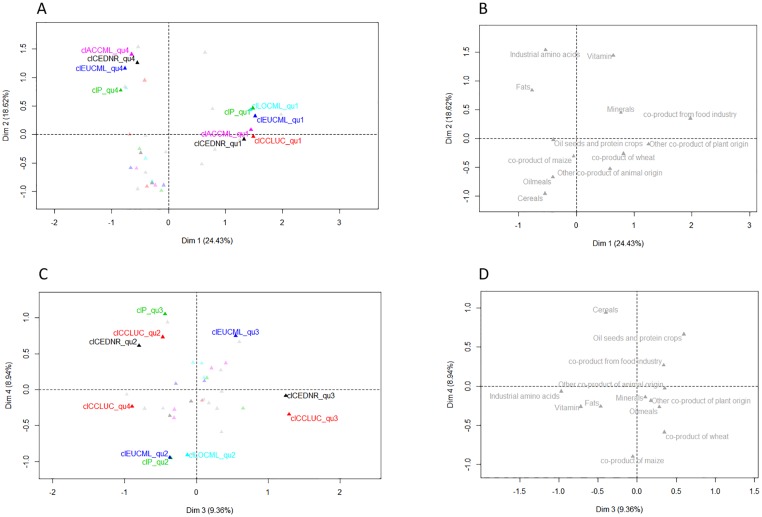
Factorial maps of the Multiple Correspondence Analysis with plotting of the active variable levels contributing more than 10% of the first 4 dimensions (A and C) and plotting of the feed-ingredient categories in the first 4 dimensions (B and D). CCLUC, AC_CML_, EU_CML_, CEDNR, PD, and LO_CML_ are climate change with land-use change, acidification, eutrophication, non-renewable cumulative energy demand, phosphorus demand, and land occupation, respectively. Levels qu1 to qu4 correspond to the 1^st^ to 4^th^ quartiles of each variable.

The MCA and HC provided a general overview of the dataset. In the MCA, the 1^st^ and 2^nd^ dimensions distinguish the 1^st^ and 4^th^ quartiles of the following environmental impacts: CCLUC, AC_ILCD_, EU_CML_, CEDNR, PD, LO_CML_ (Fig 3A). The 1^st^ quartiles of these impacts are close to each other on the factor map, which indicates that some feed ingredients in the dataset have low values for all environmental impacts. The 4^th^ quartiles of 4 of these impacts (AC_CML_, EU_CML_, CEDNR and PD) are also close to each other on the factor map, which indicates that some feed ingredients in the dataset have high values for these 4 impacts. The 1^st^ quartiles of impacts tend to be observed for co-products from the food industry, wheat co-products, co-products of animal origin, other co-products of plant origin, and minerals (Fig 3B). The 4^th^ quartiles of impacts correspond to industrial amino acids, fats and vitamins (Fig 3B).

The 3^rd^ and 4^th^ dimensions of the MCA distinguish quartiles of CCLUC, EU_CML_, CEDNR, PD, and LO_CML_ (Fig 3C). The 4^th^ dimension is positively linked to the 2^nd^ quartile of CCLUC, EU_CML_, and CEDNR, and the 3^rd^ quartile of PD, both of which characterise cereals and oilseeds and protein crops (Fig 3D).

**Fig 4:**
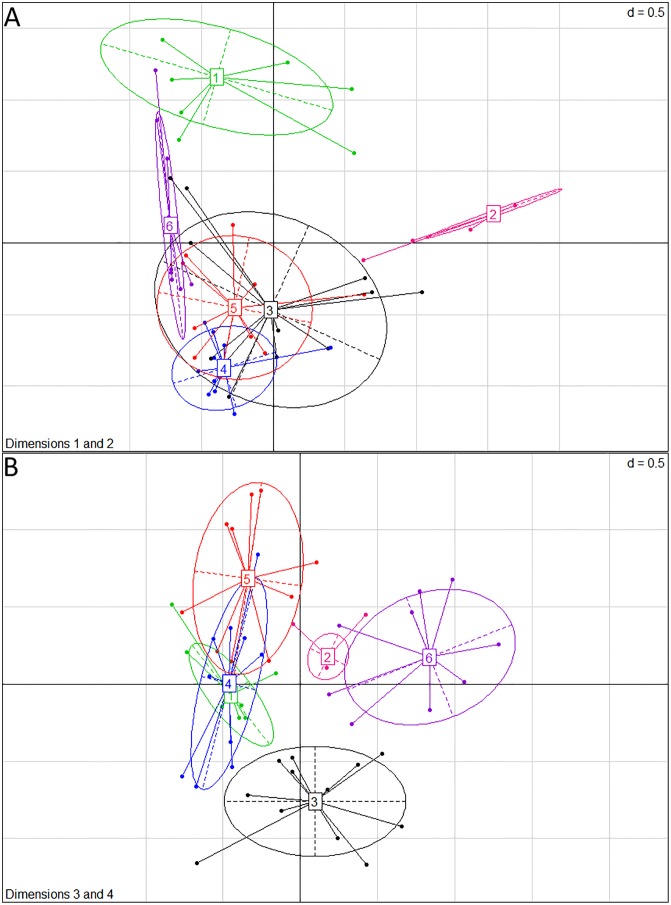
Factorial maps from Multiple Correspondence Analysis and Hierarchical Clustering, with representation of point classes of feed ingredients: Class 1—highest impacts for CCLUC, AC_CML_, EU_CML_, CEDNR, PD, and LO_CML_; Class 2 with the- lowest impacts for CCLUC, AC_CML_, EU_CML_, CEDNR, PD, and LO_CML_; Class 3—intermediate impacts for EU_CML_, PD, LO_CML_, 2^nd^ quartile impacts for AC_CML_ and 3^rd^ quartile impacts for CCLUC and CEDNR; Class 4—intermediate impacts for CCLUC, AC_CML_, EU_CML_, CEDNR, and LO_CML_; Class 5—intermediate impacts for CCLUC, CEDNR PD, and LO_CML_,; Class 6—high PD impacts and 3^rd^ quartile impacts for CCLUC, EU_CM_and CEDNR _L_. A corresponds to dimensions 1 and 2 and B to dimensions 3 and 4.

The HC built 6 classes that are separated on the 1^st^ to 4^th^ dimensions (Fig 4A and 4B). Class 1 contains 14 feed ingredients with the highest impacts for CCLUC, AC_CML_, EU_CML_, CEDNR, PD, and LO_CML_: industrial amino acids, fats and vitamins. This finding is consistent with previous LCAs on industrial amino acids [46, 47] and fats [48, 49]. Class 2 contains 14 feed ingredients with the lowest impacts for CCLUC, AC_ILCD_, EU_CML_, CEDNR, PD, LO_CML_: minerals, co-products of plant origin and co-products from the bread industry. These low impacts are partly due to the economic allocation of impacts among co-products. Class 3 contains 14 feed ingredients with intermediate impacts for AC_CML_, EU_CML_, PD, LO_CML_, (2^nd^ quartile) and CCLUC and CEDNR (3^rd^ quartile). This class contains 85% of French feed ingredients, including pea, monocalcium phosphate, sunflower meal, DDGS and maize co-products. Class 4 contains 11 feed ingredients which are cereals and imported feed ingredients with intermediate values of CCLUC, AC_CML_, EU_CML_, CEDNR, and LO_CML_. Class 5 contains 10 feed ingredients that are oilseeds and protein crops as well as cereals with intermediate values of CCLUC, CEDNR, PD, and LO_CML_. Class 6 contains oil meals and oilseeds with high PD values and 3^rd^ quartile values of CCLUC, EU_CML_ and CEDNR which come mainly from Brazil and the US.

The feed ingredient types in the ECOALIM dataset (Fig 3B and 3D) have different levels of environmental impacts. Analysis of the dataset gives some highlights for feed formulation:

- Some types have high impacts regardless of the ingredient (Class 1, e.g. amino acids) and are essential for formulating feeds. Their impacts are influenced by high energy inputs for industrial processes, such as those for amino acids, vitamins, and soya bean oil. Since these products are always incorporated in low quantities, they are not a mechanism for reducing feed impacts, except if amino acids are combined with cereals to replace soya bean meal associated with deforestation [50].
- Other types, such as co-products of plant origin and from milling and bread industries, have low impacts because of economic allocation of impacts and the low-input processes used to produce them. They can be incorporated at higher percentages into feeds to reduce the latter’s environmental burdens. Recent studies have investigated nutritional benefits of co-products [51, 52] and the subsequent potential reduction in impacts [53].
- Feed ingredient types that are incorporated in large quantities, such as cereals, oil meals and oilseeds, are distributed among classes with different impact levels (Classes 4, 5 and 6). The main processes driving their impacts include N and P fertilisation, diesel use and combustion, and deforestation for soya bean meal. Therefore, formulating feeds width constraints on potential environmental impacts should lead to substitutions among similar types of feed ingredients, which should decrease impacts.

### Comparison with other databases

**Fig 5:**
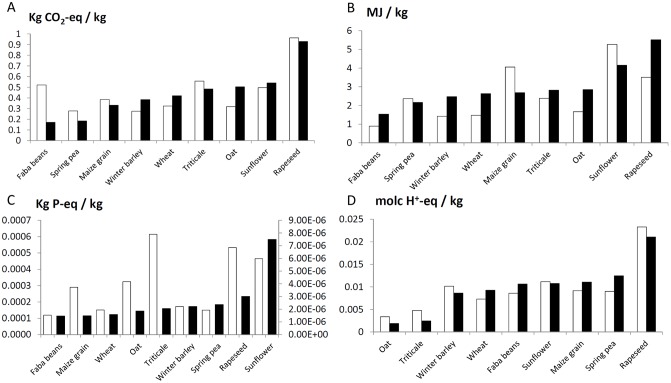
Comparison of impact assessment results per kg of feed ingredient for cereals, legumes, and oil and protein crops in the ECOALIM and Agri-Footprint databases for (A) climate change ILCD (kg CO_2_-eq), (B) non-renewable energy (MJ), (C) freshwater eutrophication ILCD (kg P-eq) and (D) acidification ILCD (molc H^+^-eq). Note that freshwater eutrophication has two axes, which correspond to (left) ECOALIM and (right) Agri-Footprint.

To our knowledge, only two other databases include French feed ingredients or crops: Agri-Footprint and ecoinvent^®^. We focused comparison on the former (Fig 5) because the latter relies mainly on Swiss data and practices for the LCIs of agricultural production and provides only a few feed-ingredient LCIs. Between ECOALIM and Agri-Footprint, rankings of CCLUC impacts of cereals, oil seeds and protein crops differ (Fig 5A) among crops (e.g. faba beans, oat, winter barley). Nevertheless, some consistency exists: rapeseed has the highest impact and sunflower and triticale have similar values in both datasets. Regarding CEDNR (Fig 5B), except for the lowest impact observed for faba beans in both datasets, ECOALIM and Agri-Footprint show different values for most crops: winter barley, wheat, maize grain, oat, rapeseed, and sunflower. Consequently, the ranking of the crops differs between the two datasets.

The values of freshwater EU (Fig 5C) are much higher (x100) in ECOALIM than in Agri-Footprint, and the ranking of crops is completely different. P leaching is calculated as a fixed proportion of P applied in Agri-Footprint, whereas the SALCA-P model used in ECOALIM accounts for the P applied (mineral and organic), crop duration (time between harvest of the considered crop and the preceding crop), soil erosion, and allocation of fertilisation among crops in the same rotation. AC impact (Fig 5D) is consistent between both datasets. In each case, rapeseed has the highest value, and oat and triticale have the lowest ones values. The other crops have moderate differences in value between datasets, rendering their rankings different.

When formulating feeds based on environmental impacts, these databases would yield different feeds. Feed formulation is an optimisation problem that relies on linear programming with nutritional constraints. Consequently, when minimising environmental impacts of feeds at a given nutritional level, incorporation of feed ingredients will follow the impact ranking of feed ingredients. Therefore, feed manufacturers need the most accurate data on environmental impacts that account for specific crop-management practices, data provided for the French context by the ECOALIM dataset.

### Use, maintenance and updating of the dataset

The ECOALIM dataset is the animal-nutrition subset of the AGRIBALYSE^®^ database. Therefore, it is also available in the Agribalyse^®^ v1.3 database in SimaPro^®^, with the following name format for processes:

- for unprocessed feed ingredients: “<Feed ingredient name>, <type of production>, <crop-management practice>, animal feed, <LCA perimeter> /FR U”, e.g. Feed barley grain, conventional, national average, animal feed, at farm gate/FR U.
- for processed feed ingredients: “<Feed ingredient name>, animal feed, <LCA perimeter> /FR U”, e.g. Sunflower meal, with low dehulling, animal feed, at transformation plant/FR U.

**Fig 6:**
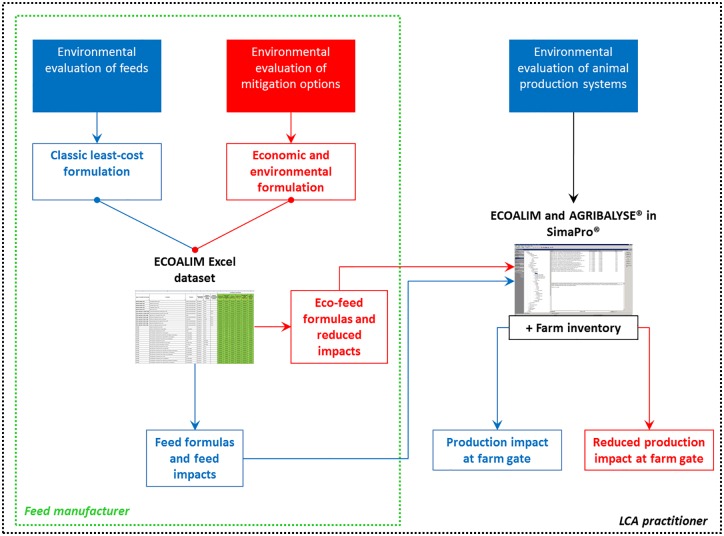
Diagram of potential uses of the ECOALIM dataset. The blue pathway corresponds to the methodology classically used when estimating environmental impacts of feeds and animal products at the farm gate. The red pathway illustrates the methodology used when evaluating strategies to mitigate environmental impacts of feeds and animal products at the field gate.

The ECOALIM dataset can be used to estimate environmental impacts of feeds, animal production systems and mitigation options (Fig 6).

Both feed manufacturers and LCA practitioners can formulate feeds according to the classic least-cost approach and calculate the environmental impacts of the resulting feeds using the ECOALIM dataset in Excel. To investigate mitigation options, they can also formulate “eco-feeds” based on both economic and environmental approaches by using the ECOALIM dataset in Excel in formulation software. In an initial application, eco-feeds formulated for pig production had impacts 6–15% (assuming current availability of feed ingredients) and 12–26% (assuming improved availability of some feed ingredients) lower than those of the least-cost formulation, depending on the impact considered [54]. The ECOALIM dataset in the AGRIBALYSE^®^ database in SimaPro^®^ can be used by LCA practitioners in more detailed farm LCIs to assess environmental impacts of animal products at the farm gate using either least-cost feed or eco-feed formulas. For these applications, the ECOALIM dataset offers standardised, updated and reviewed data. It covers a range of feed ingredients that was not previously available in AGRIBALYSE^®^ and is, to date, the only dataset that relies on reliable foreground data representative for France, collected from multiple surveys following a common framework. To our knowledge, ECOALIM is the first dataset available for feed ingredient LCIs and impacts that provides all feed ingredients used in conventional livestock production in France and specific LCIs for systematic cover cropping, systematic organic fertilisation, and introduction of a protein crop. It also applies to other European countries for several imported feed ingredients, as well as for French feed ingredients which are exported. This is a major added-value of the dataset because it provides reliable data to non-experts in LCA who may find it very difficult to gather properly data from various databases and publications [20].

Incorporation of the ECOALIM dataset into the AGRIBALYSE^®^ database allows future updating of the LCIs and the addition of new feed ingredients, such as fish meal and oil for aquaculture production. Furthermore, integration of the AGRIBALYSE^®^ database into SimaPro^®^ software ensures appropriate maintenance of the data and wider distribution among LCA practitioners.

## Conclusion

The ECOALIM dataset provides life cycle impacts of feed ingredients used in French livestock production based on a standardised methodology and homogeneous foreground and background data. This approach avoids double-counting of environmental burdens. The approach was developed to be consistent with the AGRIBALYSE^®^ database, which will allow future use of the ECOALIM dataset for feed formulation based on environmental impacts and environmental labelling of animal products. The ECOALIM dataset relies on representative and recent data that cover a wide range of production practices in France. Built with organisations such as feed manufacturers, raw material producers and R&D institutes, this dataset contains feed ingredients necessary to formulate complete feeds and several French feed ingredients for which no data were available before (i.e. sorghum, flaxseed, and soya bean). The ECOALIM dataset will allow feed formulas to be developed which incorporate nutritional, economic and environmental constraints. This new approach to designing diets needs further research to evaluate its potential as an option for mitigating livestock-related impacts. It would be interesting to conduct a similar study in other countries with the same degree of accuracy in LCI data of feed ingredients. The framework developed in the AGRIBALYSE^®^ database and ECOALIM dataset is adequate to further develop such databases.

## References

[1] Gerber PJ, Steinfeld H, Henderson B, Mottet A, Opio C, Dijkman J, et al. Tackling climate change through livestock: a global assessment of emissions and mitigation opportunities. Report. Food and Agriculture Organization of the United Nations, 2013.

[2] Billen G, Lassaletta L, Garnier J. A vast range of opportunities for feeding the world in 2050: trade-off between diet, N contamination and international trade. Environmental Research Letters. 2015;10(2).

[3] FAO. World livestock 2011-Livestock in food security. Rome, Italy: Food and Agriculture Organization of the United Nations (FAO), 2011.

[4] Steinfeld H, Gerber P, Wassenaar T, Castel V, Rosales M, Haan Cd. Livestock's long shadow: environmental issues and options. Rome Italy: Food and Agriculture Organization of the United Nations (FAO), 2006 978-92-5-105571-7.

[5] Environmental Management—Life Cycle Assessment—Principles and Framework. EN ISO 14040, (2006).

[6] van der Werf HMG, Petit J. Evaluation of the environmental impact of agriculture at the farm level: a comparison and analysis of 12 indicator-based methods. Agricult Ecosyst Environ. 2002;93(1/3):131–45.

[7] de Vries M, de Boer IJM. Comparing environmental impacts for livestock products: A review of life cycle assessments. Livest Sci. 2010;128(1–3):1–11.

[8] Basset-Mens C, van der Werf HMG. Scenario-based environmental assessment of farming systems: the case of pig production in France. Agr Ecosyst Environ. 2005;105(1–2):127–44.

[9] Dourmad JY, Ryschawy J, Trousson T, Bonneau M, Gonzalez J, Houwers HWJ, et al Evaluating environmental impacts of contrasting pig farming systems with life cycle assessment. Animal. 2014;8(12):2027–37. 10.1017/S1751731114002134 25170767

[10] Boggia A, Paolotti L, Castellini C. Environmental impact evaluation of conventional, organic and organic-plus poultry production systems using life cycle assessment. World's Poultry Science Journal. 2010;66(01):95–114.

[11] Prudêncio da Silva V, van der Werf HMG, Soares SR, Corson MS. Environmental impacts of French and Brazilian broiler chicken production scenarios: An LCA approach. J Environ Manag. 2014;133:222–31. 10.1016/j.jenvman.2013.12.011 24388925

[12] Leinonen I, Williams AG, Wiseman J, Guy J, Kyriazakis I. Predicting the environmental impacts of chicken systems in the United Kingdom through a life cycle assessment: Broiler production systems. Poultry Science. 2012;91(1):8–25. 10.3382/ps.2011-01634 22184424

[13] Leinonen I, Williams AG, Wiseman J, Guy J, Kyriazakis I. Predicting the environmental impacts of chicken systems in the United Kingdom through a life cycle assessment: Egg production systems. Poultry Science. 2012;91(1):26–40. 10.3382/ps.2011-01635 22184425

[14] MacLeod M, Gerber P, Mottet A, Tempio G, Falcucci A, Opio C, et al Greenhouse gas emissions from pig and chicken supply chains—a global life cycle assessment. Rome, Italy: Food and Agriculture Organization of the United Nations (FAO), 2013.

[15] FAO. Greenhouse Gas Emissions from the Dairy Sector: A Life Cycle Assessment. Rome, Italy: Food and Agriculture Organization of the United Nations (FAO), 2010.

[16] Nguyen TLT, Hermansen JE, Mogensen L. Environmental consequences of different beef production systems in the EU. J Cleaner Prod. 2010;18(8):756–66.

[17] Garcia-Launay F, van der Werf HMG, Nguyen TTH, Le Tutour L, Dourmad JY. Evaluation of the environmental implications of the incorporation of feed-use amino acids in pig. production using Life Cycle Assessment. Livest Sci. 2014;161:158–75.

[18] Nguyen TTH, Bouvarel I, Ponchant P, van der Werf HMG. Using environmental constraints to formulate low-impact poultry feeds. J Cleaner Prod. 2012;28:215–24.

[19] Prudêncio da Silva V, van der Werf HMG, Spies A, Soares SR. Variability in environmental impacts of Brazilian soybean according to crop production and transport scenarios. J Environ Manage. 2010;91(9):1831–9. 10.1016/j.jenvman.2010.04.001 20452717

[20] Bertoluci G, Masset G, Gomy C, Mottet J, Darmon N. How to Build a Standardized Country-Specific Environmental Food Database for Nutritional Epidemiology Studies. Plos One. 2016;11(4). 10.1371/journal.pone.0150617 PMC4824438 27054565

[21] Durlinger B, Tyszler M, Scholten J, Broekema R, Blonk H. Agri-footprint; A life cycle inventory database covering food and feed production and processing. In: Schenck R, Huizen D, editors. Proceedings of the 9th International Conference on Life Cycle Assessment in the Agri-Food Sector; San Francisco, USA.2014. p. 310–7.

[22] Nemecek T, Kägi T. Life cycle inventories of Swiss and European agricultural production systems. Final report ecoinvent report v2.0, n°15. Agroscope Reckenholz-Taenikon Research station ART. Swiss Centre for Life Cycle Inventories. Zurich and Dübendorf, Switzerland: 2007.

[23] Colomb V, van der werf HMG, Gac A, Mousset J, Tailleur A, Wilfart A. How to reconcile eco-design and eco-labelling in LCI database construction? AGRIBALYSE experience and links with database harmonization initiatives. 10th International Conference on Life Cycle Assessment of Food 2016, LCA Food 2016; Dublin, Ireland.2016.

[24] Koch P, Salou T. AGRIBALYSE(R): Methodological report—Version 1.2. Angers. France: ADEME, 2015.

[25] EMEP/EEA. Air pollutant emission inventory guidebook—Technical Report N°12. Copenhagen, Denmark: European Environment Agency (EEA), 2013.

[26] Agreste. Enquête sur les pratiques culturales 2006. http://agreste.agriculture.gouv.fr/publications/chiffres-et-donnees/article/enquete-sur-les-pratiques.

[27] Agreste. Statistique Agricole Annuelle 2005–2012. http://www.agreste.agriculture.gouv.fr/enquetes/statistique-agricole-annuelle-saa/.

[28] UNIP. Statistiques France protéagineux 2005–2009. http://www.unip.fr/marches-et-reglementations/statistiques-france/surfaces-rendements-et-productions.html.

[29] Roeder M, Thornley P, Campbell G, Bows-Larkin A. Emissions associated with meeting the future global wheat demand: A case study of UK production under climate change constraints. Environmental Science & Policy. 2014;39:13–24.

[30] Feed Grains: Yearbook Tables [Internet]. USDA Economic Research Service. 2014. http://www.ers.usda.gov/data-products/feed-grains-database/feed-grains-yearbook-tables.aspx.

[31] Farm Financial and Crop Production Practices [Internet]. USDA Economic Research Service. 2014. http://www.ers.usda.gov/data-products/arms-farm-financial-and-crop-production-practices/tailored-reports-farm-structure-and-finance.aspx#P369773ec09fe4390a25fcbff02a2728a_7_66iT0R0T0R0x2.

[32] Quick Stats [Internet]. USDA National Agricultural Statistics Service. 2010. http://quickstats.nass.usda.gov/results/3C1C82DB-0009-3F07-91DE-1D1907917ABD.

[33] State Statistics Service of Ukraine. Agriculture of Ukraine—Statistical Yearbook. Kiev, Ukraine: 2012.

[34] FAOStat Production statistics [Internet]. Food and Agriculture Organization of the United Nations (FAO). 2013. http://faostat.fao.org/site/291/default.aspx.

[35] Boissy J, Aubin J, Drissi A, van der Werf HMG, Bell GJ, Kaushik SJ. Environmental impacts of plant-based salmonid diets at feed and farm scales. Aquaculture. 2011;321(1–2):61–70.

[36] Weidema BP, Bauer C, Hischier R, Mutel C, Nemecek T, Reinhard J, et al. The ecoinvent database: Overview and methodology, Data quality guideline for the ecoinvent database version 3, www.ecoinvent.org. 2013.

[37] IPCC. Emissions from livestock and manure management IPCC guidelines for National Greenhouse Gas Inventories. 4(10): IPCC; 2006 p. 87

[38] Foster GR. Revised Universal Soil loss equation—Version 2 (RUSLE 2). Report. Washington, D.C.: USDA, Agricultural Research Service; 2005.

[39] COMIFER. Lessivage des nitrates en systèmes de cultures annuelles Diagnostic de risque et propositions de gestion de l'interculture. COMIFER, editor. Puteaux, France 2001.

[40] Tailleur A, Cohan JP, Laurent F, Lellahi A. A simple model to assess nitrate leaching from annual crops for life cycle assessment at different spatial scales. In: Corson MS, van der Werf HMG, editors. Proceedings of the 8th International Conference on Life Cycle Assessment in the agri-food sector (LCA Food 2012), 1–4 October 2012; Saint-Malo, France: INRA Rennes, France; 2012. p. 903–4.

[41] Freiermuth R. Modell zur Berechnung der Schwermetallflüsse in der Landwirstschaftlichen Okobilanz. Zurich, Switzerland: Agroscope FAL Reckenholz, 2006.

[42] SOGREAH. Bilan des flux de contaminants entrant sur les sols agricoles de France métropolitaine. Angers, France: ADEME, 2007.

[43] JRC. Characterisation factors of the ILCD recommanded life cycle impact assessment methods database and supporting information. European Commission, Joint Research Centre, Institute for Environment and Sustainability, 2012.

[44] SimaPro Database Manual [Internet]. 2015. https://www.pre-sustainability.com/download/DatabaseManualMethods.pdf.

[45] R Development Core Team. R: A language and environment for statistical computing. Vienna, Austria: R Foundation for Statistical Computing; 2015.

[46] Meul M, Ginneberge C, Van Middelaar CE, de Boer IJM, Fremaut D, Haesaert G. Carbon footprint of five pig diets using three land use change accounting methods. Livest Sci. 2012;149(3):215–23. 10.1016/j.livsci.2012.07.012.

[47] Marinussen M, Kool A. Environmental impacts of synthetic amino acid production. Netherlands: Blonk Milieu Advies BV, 2010.

[48] Dalgaard R, Schmidt J, Halberg N, Christensen P, Thrane M, Pengue WA. LCA of soybean meal. The International Journal of Life Cycle Assessment. 2007;13(3):240–54.

[49] Schmidt JH. Life cycle assessment of rapeseed oil and palm oil: Ph.D. thesis, Part 3: life cycle inventory of rapeseed oil and palm oil. Denmark: Aalborg University; 2007.

[50] Monteiro ANTR, Garcia-Launay F, Brossard L, Wilfart A, Dourmad JY. Effect of feeding strategy on environmental impacts of pig fattening in different contexts of production: evaluation through Life Cycle Assessment. Journal of Animal Science. 2016;in press. 10.2527/jas.2016-0529 27898927

[51] Cozannet P, Lessire M, Gady C, Metayer JP, Primot Y, Skiba F, et al Energy value of wheat dried distillers grains with solubles in roosters, broilers, layers, and turkeys. Poultry Science. 2010;89(10):2230–41. 10.3382/ps.2010-00833 20852114

[52] Cozannet P, Primot Y, Gady C, Metayer JP, Lessire M, Skiba F, et al Energy value of wheat distillers grains with solubles for growing pigs and adult sows. Journal of Animal Science. 2010;88(7):2382–92. 10.2527/jas.2009-2510 20228235

[53] Zanten HHE, Mollenhorst H, Vries JW, Middelaar CE, Kernebeek HRJ, Boer IJM. Assessing environmental consequences of using co-products in animal feed. The International Journal of Life Cycle Assessment. 2013;19(1):79–88.

[54] Garcia-Launay F, Wilfart A, Dusart L, Nzally C, Gaudré D, Espagnol S. Multi-objective formulation is an efficient methodology to reduce environmental impacts of pig feeds. 10th International Conference on Life Cycle Assessment of Food 2016, LCA Food 2016; 19–21 October 2016; Dublin, Ireland2016.

